# Peptriever: a Bi-Encoder approach for large-scale protein–peptide binding search

**DOI:** 10.1093/bioinformatics/btae303

**Published:** 2024-05-06

**Authors:** Roni Gurvich, Gal Markel, Ziaurrehman Tanoli, Tomer Meirson

**Affiliations:** Davidoff Cancer Center, Rabin Medical Center-Beilinson Hospital, Petah Tikva 49100, Israel; Davidoff Cancer Center, Rabin Medical Center-Beilinson Hospital, Petah Tikva 49100, Israel; Faculty of Medicine, Tel Aviv University, Tel-Aviv 6997801, Israel; Samueli Integrative Cancer Pioneering Institute, Rabin Medical Center-Beilinson Hospital, Petah Tikva, Israel; Institute for Molecular Medicine Finland (FIMM), HiLIFE, University of Helsinki, Helsinki 00290, Finland; Davidoff Cancer Center, Rabin Medical Center-Beilinson Hospital, Petah Tikva 49100, Israel; Faculty of Medicine, Tel Aviv University, Tel-Aviv 6997801, Israel; Samueli Integrative Cancer Pioneering Institute, Rabin Medical Center-Beilinson Hospital, Petah Tikva, Israel

## Abstract

**Motivation:**

Peptide therapeutics hinge on the precise interaction between a tailored peptide and its designated receptor while mitigating interactions with alternate receptors is equally indispensable. Existing methods primarily estimate the binding score between protein and peptide pairs. However, for a specific peptide without a corresponding protein, it is challenging to identify the proteins it could bind due to the sheer number of potential candidates.

**Results:**

We propose a transformers-based protein embedding scheme in this study that can quickly identify and rank millions of interacting proteins. Furthermore, the proposed approach outperforms existing sequence- and structure-based methods, with a mean AUC-ROC and AUC-PR of 0.73.

**Availability and implementation:**

Training data, scripts, and fine-tuned parameters are available at https://github.com/RoniGurvich/Peptriever. The proposed method is linked with a web application available for customized prediction at https://peptriever.app/.

## 1 Introduction

Peptides and proteins are fundamental biological molecules distinguished by differences in size and functional complexity. Peptides consist of relatively short chains of amino acids, that are typically interconnected by peptide bonds. In contrast, proteins are characterized by extensive, unbranched polypeptide chains with a molecular mass exceeding 10 000 Daltons. Protein–peptide interactions play a crucial role in various cellular processes, making their precise elucidation essential for the development of peptide-based therapeutics (Lei *et al.* 2021), advancement in biosensing technologies (Caporale *et al.* 2021), and the design of vaccines (Di Natale *et al.* 2020).

The experimental identification of protein–peptide interactions poses challenges in terms of cost and time (Johansson-Åkhe *et al.* 2019). Consequently, computational methodologies have emerged as valuable alternative tools capable of unleashing the potential of peptides as innovative agents in medicine and biology. (Audie and Swanson 2013). Several computational methods have been developed to predict protein–protein or protein–peptide interactions. Notable examples include InterPep2 (Johansson-Åkhe *et al.* 2020), PIPER-FlexPepDock (PFPD) (Alam *et al.* 2017), CABS-dock (Kurcinski *et al.* 2015) and CAMP (Lei *et al.* 2021). AlphaFold-Multimer, an extension of AlphaFold2, performs better than state-of-the-art methods for protein–peptide complex modeling and interaction prediction (Evans *et al.* 2021, Ko and Lee 2021, Johansson-Åkhe and Wallner 2022). The existing methods primarily estimate the binding probability between protein and peptide pairs. However, for a specific peptide sequence without a corresponding protein, it is challenging to identify the proteins it binds as there may be millions of candidates.

By carefully examining the pros and cons of the currently available methods, we developed Peptriever. This user-friendly web application leverages the power of transformer-based architecture on its backend, enabling lightning-fast peptide binding searches across millions of proteins. Peptriever harnesses an approximate nearest neighbor index populated with high-dimensional vector representations (embeddings) of the proteins. These embeddings are derived through the use of two transformers jointly trained for this purpose. Herein we present the Peptriever's architecture, training data, and results.

## 2 Materials and methods

### 2.1 Architecture and training

Peptriever employed a BERT-like architecture (Devlin *et al.* 2018, https://arxiv.org/pdf/1810.04805) coupled with a byte-pair encoding tokenizer (Sennrich *et al.* 2015, https://arxiv.org/abs/1508.07909) to train a binding embedding model. We used byte-pair encoding instead of character-wise tokenizer, as it allows efficient representation of subword units, improves out-of-vocabulary word handling, and balances granularity, making it a versatile choice especially for bioinformatics applications (Filipavicius *et al.* 2020, https://arxiv.org/abs/2012.03084). Additional details regarding Peptriever, are presented in the Supplementary Material.

The training process for Peptriever comprised two stages: unsupervised pre-training and supervised fine-tuning.

In the first stage, two separate models were trained, one for proteins and another for peptides. Following the footsteps of Protein BERT (Brandes *et al.* 2022), the models were trained using a Masked Language Modeling (MLM) task (Sennrich et al. 2015, https://arxiv.org/abs/1508.07909). During training, the model received a tokenized input sequence for each sample batch with a randomly chosen subset of masked tokens. Subsequently, the model learned to replace these masked tokens with their original counterparts, leveraging information from the nonmasked tokens on both sides of the sequence. Prior to the pre-training, the tokenizer was trained on the same dataset with its vocabulary size set to 1024.

It has been shown that two transformer models jointly trained with a contrastive loss function can learn to map different inputs to the same embedding space even when the inputs come from different modalities, such as text and image (Radford *et al.* 2021). Similar architecture called Bi-Encoder (Jung *et al.* 2021) was presented for neural ranking to improve text search.

Therefore, the two pre-trained models were jointly fine-tuned in the second stage in a similar way; the fine-tuning process aimed to minimize the distance in the embedding space between matched protein–peptide sets while maximizing the distance between a protein and all nonmatching peptides. Additionally, the MLM loss was added to enhance the generalization of the embeddings.

### 2.2 Training and validation

To conduct unsupervised pre-training, 202 738 PDB files were obtained from the RCSB Protein Data Bank (Berman *et al.* 2000), and protein sequences were extracted into a dataset. The dataset included 40 105 genes with 159 907 unique sequences from 6768 species. Sequences <30 amino acids were considered peptides, and sequences >50 amino acids were considered proteins.

To enable supervised fine-tuning, we integrated several databases of protein–peptide interactions across multiple species. Our data was sourced from PepBDB (Wen *et al.* 2019), Propedia (Martins *et al.* 2021), and YAPP-Cd(Park 2021), all of which are based on experimentally verified interactions with 3D structures. After removing duplicates and discarding structure information, the resulting training dataset included 16 370 unique pairs of protein–peptide sequences.

To prevent data leakage caused by near-identical sequences in different training partitions, we used the gene association of the protein and the peptide to assign these to the train, validation, and test partitions. For instance, if a protein was associated with gene A and a peptide was associated with gene B, any other combinations of protein–peptide pairs in the training set sharing the same gene A and gene B associations were grouped within the same training partition.

For evaluation, we used the test set utilized by Johansson-Åkhe and Wallner (2022). To avoid data leaks, overlapping protein–peptide sequence sets from the test set were excluded during fine-tuning.

### 2.3 Evaluation

The quality of the embeddings was benchmarked against CAMP, InterPep2, and various versions of AlphaFold-Multimer, as previously reported in (Johansson-Åkhe and Wallner 2022). The test set defined protein–peptide interaction prediction as a binary classification task comprising 112 positive and 560 negative interactions. The performance of the model was evaluated by computing the area under the Precision–Recall curve (AUP-PR) and the area under the Receiver Operating Characteristic curve (AUC-ROC). The negated and normalized distance between the embeddings of each pair was used as the predicted probability, as shown in the following equation.

$$P_{\mathrm{bind}}=1-\;\mathrm{dist}/\max(\mathrm{dist})$$

where the max(dist) value is the maximal distance observed for all positive and negative pairs. It is calculated once and treated as a scaling parameter (i.e. distance scores for all new proteins are scaled using this parameter).

## 3 Results

**Figure 1.**
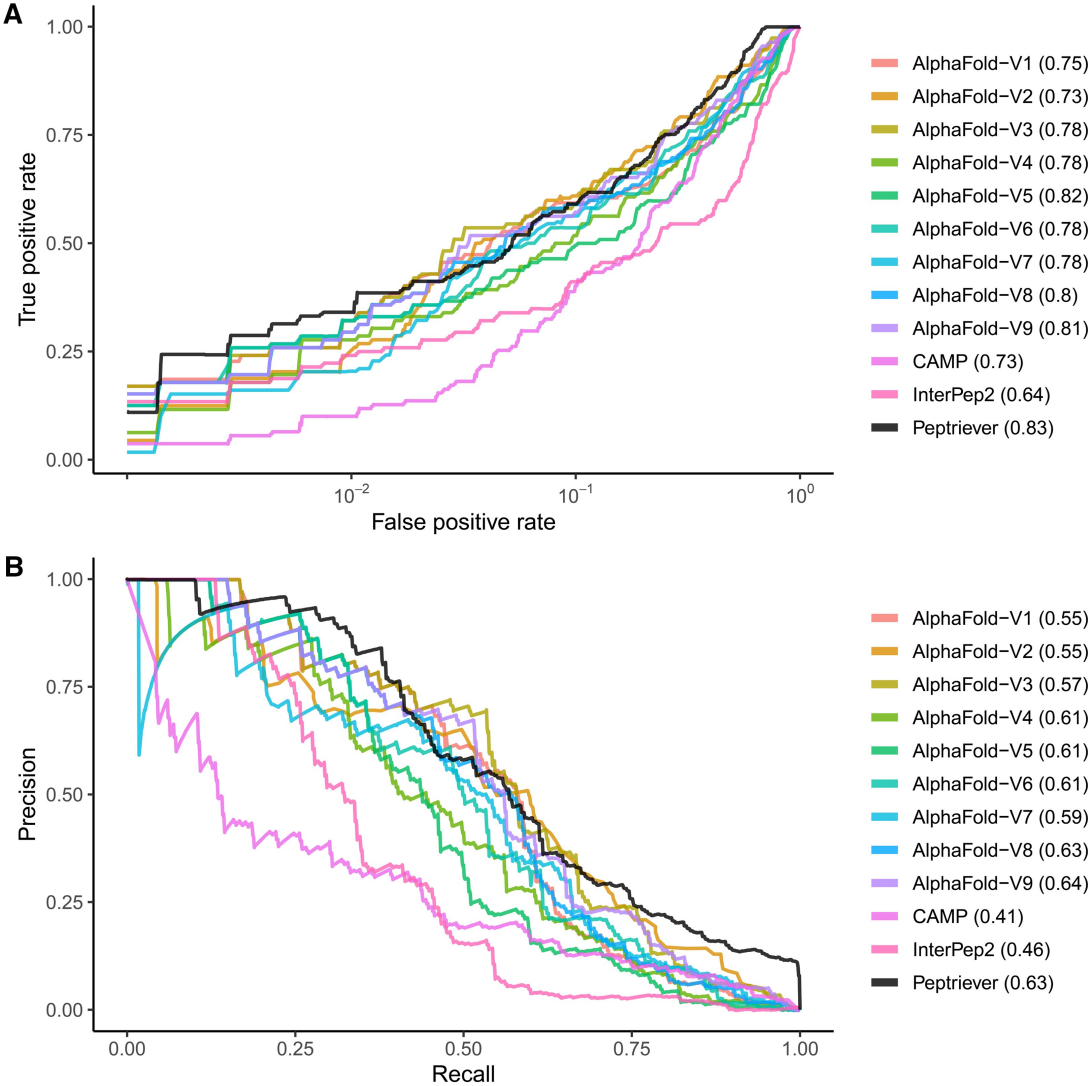
Performance measures for peptide–protein interaction prediction. (A) Receiver–operator curve (ROC) with the *x*-axis in log-scale. (B) Precision–recall (PR) curve. The values in the legends correspond to the area under the ROC or PR curves. Curve coordinates from CAMP, InterPep2, and the various versions AlphaFold-Multimer were extracted from Johansson-Åkhe *et al.* using WebPlotDigitizer.

The performance comparison between the proposed Peptriever and other methods is shown in Fig. 1. Peptriever embedding achieved an AUC-ROC of 0.83, which was higher than the best-performing version of AlphaFold-Multimer (v9) with 0.81, CAMP with 0.73, and InterPep2 with 0.64. Furthermore, the model’s AUC-PR, which is a more reasonable measure than AUC-ROC given the higher number of samples with negative versus positive interactions in our study, was 0.63. This AUC-PR outperformed all versions of AlphaFold-Multimer, except v8 and v9, which achieved comparable scores of 0.63 and 0.64, respectively. It also surpassed the scores of CAMP (0.41) and InterPep2 (0.46). Collectively, the Peptriever embeddings exhibited a mean AUC-ROC and AUC-PR of 0.73, which was higher than all other models, including AlphaFold-Multimer v9, where the mean was 0.725. Performance improvement was possibly due to the BERT-based architecture combined with a contrastive loss function.

Notably, Peptriever and Alphafold or Alphafold-multimer cannot be directly compared with respect to speed, since in Peptriever, the embeddings are pre-computed. However, from the perspective of the end-user, Peptriever only requires a couple of seconds to identify/rank proteins for the entire proteome, whereas predicting a single complex using Alphafold or Alphafold-multimer requires minutes to hours (Bryant *et al.* 2022, Tsaban *et al.* 2022).

### 3.1 Application

The embeddings are stored in a vector database. Upon user input of a sequence, the database is searched for proteins with the highest binding probabilities, either across all species or a particular species as specified by the user.

### 3.2 Limitation

When training BERT-like models, sequences are adjusted to a fixed length through truncation or padding. We set the peptide and protein model input to 30 and 300 tokens, respectively. These lengths were chosen to encompass >90% of the proteins in the training set, ensuring that all the peptides were included. However, the performance may be suboptimal for proteins with longer sequences if important information is located toward the end, as it would be truncated.

## 4 Conclusion

Peptriever provides a user-friendly web interface, allowing users to input peptide sequences and retrieve protein binding scores across any number of proteins. The current version of Peptriever is limited to peptide search; however, in the next version, users will be able to input protein sequences and look for peptides that would bind them. Peptriever can be a useful tool for translational researchers, particularly for developing peptide therapeutics and in vaccine design.

## Supplementary Material

btae303_Supplementary_Data

## Data Availability

Training data, scripts, and fine-tuned parameters are available at https://github.com/RoniGurvich/Peptriever. The proposed method is linked with a web application available for customized prediction at https://peptriever.app/.

## References

[btae303-B1] Alam N , Goldstein O, Xia B et al High-resolution global Peptide–Protein docking using fragments-based PIPER-FlexPepDock. PLoS Comput Biol 2017;13:e1005905. 29281622 10.1371/journal.pcbi.1005905 PMC5760072

[btae303-B2] Audie J , Swanson J. Advances in the prediction of protein–peptide binding affinities: implications for peptide‐based drug discovery. Chem Biol Drug Des 2013;81:50–60. 23066895 10.1111/cbdd.12076

[btae303-B3] Berman HM , Westbrook J, Feng Z et al The protein data bank. Nucleic Acids Res 2000;28:235–42. 10592235 10.1093/nar/28.1.235 PMC102472

[btae303-B4] Brandes N , Ofer D, Peleg Y et al ProteinBERT: a universal deep-learning model of protein sequence and function. Bioinformatics 2022;38:2102–10. 35020807 10.1093/bioinformatics/btac020 PMC9386727

[btae303-B5] Bryant P , Pozzati G, Zhu W et al Predicting the structure of large protein complexes using AlphaFold and Monte Carlo tree search. Nat Commun 2022;13:6028. 36224222 10.1038/s41467-022-33729-4 PMC9556563

[btae303-B6] Caporale A , Adorinni S, Lamba D et al Peptide–protein interactions: from drug design to supramolecular biomaterials. Molecules 2021;26:1219. 33668767 10.3390/molecules26051219 PMC7956380

[btae303-B22] Devlin J, Chang M.-W, Lee, K et al ‘Bert: Pre-Training of Deep Bidirectional Transformers for Lan guage Understanding’, ArXiv Preprint ArXiv:1810.04805, 2018.

[btae303-B7] Di Natale C , La Manna S, De Benedictis I et al Perspectives in peptide-based vaccination strategies for syndrome coronavirus 2 pandemic. Front Pharmacol 2020;11:578382. 33343349 10.3389/fphar.2020.578382 PMC7744882

[btae303-B21] Evans R, O'Neill M, Pritzel A, et al ‘Protein Complex Pre diction with AlphaFold-Multimer’, BioRxiv, 2021, 2010–21.

[btae303-B24] Filipavicius M, et al “Pre-training protein language models with label-agnostic binding pairs enhances performance in downstream tasks.” arXiv preprint arXiv:2012.03084 (2020).

[btae303-B9] Johansson-Åkhe I , Mirabello C, Wallner B. InterPep2: global peptide–protein docking using interaction surface templates. Bioinformatics 2020;36:2458–65. 31917413 10.1093/bioinformatics/btaa005 PMC7178396

[btae303-B10] Johansson-Åkhe I , Mirabello C, Wallner B. Predicting protein–peptide interaction sites using distant protein complexes as structural templates. Sci Rep 2019;9:4267. 30862810 10.1038/s41598-019-38498-7 PMC6414505

[btae303-B11] Johansson-Åkhe I , Wallner B. Improving peptide–protein docking with AlphaFold-multimer using forced sampling. Front Bioinform 2022;2:959160. 36304330 10.3389/fbinf.2022.959160 PMC9580857

[btae303-B12] Jung E , Choi J, Rhee W. *Semi-Siamese bi-encoder neural ranking model using lightweight fine-tuning*. arXiv preprint arXiv:2110.14943, 2021.

[btae303-B13] Ko J , Lee J. Can AlphaFold2 predict protein–peptide complex structures accurately? bioRxiv, 10.1021/acs.jpcb.2c04346, 2021, preprint: not peer reviewed.

[btae303-B14] Kurcinski M , Jamroz M, Blaszczyk M et al CABS-dock web server for the flexible docking of peptides to proteins without prior knowledge of the binding Site. Nucleic Acids Res 2015;43:W419–24. 25943545 10.1093/nar/gkv456 PMC4489223

[btae303-B15] Lei Y , Li S, Liu Z et al Deep-learning framework for Multi-Level peptide–protein interaction prediction. Nat Commun 2021;12:5465. 34526500 10.1038/s41467-021-25772-4 PMC8443569

[btae303-B16] Martins PM , Santos LH, Mariano D et al Propedia: a database for protein–peptide identification based on a hybrid clustering algorithm. BMC Bioinformatics 2021;22:1–20. 33388027 10.1186/s12859-020-03881-z PMC7776311

[btae303-B17] Park J-S. YAPP-CD: yet another protein–peptide complex database. bioRxiv, 2021, preprint: not peer reviewed.

[btae303-B18] Radford A , Kim JW, Hallacy C et al *Learning transferable visual models from natural language supervision*. arXiv preprint arXiv:2103.00020 2021, 8748–63.

[btae303-B23] Sennrich R, Haddow B, and Birch, A. ‘Neural Machine Translation of Rare Words with Subword Units’, ArXiv Preprint ArXiv:1508.07909, 2015.

[btae303-B19] Tsaban T , Varga JK, Avraham O et al Harnessing protein folding neural networks for peptide–protein docking. Nat Commun 2022;13:176. 35013344 10.1038/s41467-021-27838-9 PMC8748686

[btae303-B20] Wen Z , He J, Tao H et al PepBDB: a comprehensive structural database of biological peptide–protein interactions. Bioinformatics 2019;35:175–7. 29982280 10.1093/bioinformatics/bty579

