# Supplementary material for: Peptriever: a Bi-Encoder approach for large-scale protein–peptide binding search
Source: Bioinformatics. 2024 May 6;40(5):btae303. doi: 10.1093/bioinformatics/btae303 (PMC11112044; doi:10.1093/bioinformatics/btae303)
Supplement: btae303_Supplementary_Data [file btae303_supplementary_data.pdf]

Peptriever: A Bi-Encoder approach for large-scale protein-peptide binding search

Roni Gurvich et al.

<u>Supplementary Materials</u>

Methods

1. Calculating the distance in the embedding space

We used Euclidean distance as a distance function in the embedding space:

$$d(P,Q) = \sqrt{\sum_{i=1}^{n} p_i - q_i}$$

P and Q are the embedding vectors, the distance is defined for any given two vectors $\mathrm{P}, \mathrm{Q} \in \mathbb{R}^{\mathrm{n}}$

2. Loss of function formulation

The loss function in the second training stage (the fine-tuning stage) was defined as:

$$Loss = L_{margin} + 0.1 * \left(\frac{L1_{Ce} + L2_{Ce}}{2}\right)$$

The cross-entropy losses ($\mathrm{L1_{CE}}, \mathrm{L2_{Ce}}$) are the same as in the pretraining stage. For each token, the cross entropy between the predicted probability distribution and the actual token (the one not encoded) was calculated and then averaged across all tokens in the sequence. As two sequences and two embedding models were used in the fine-tuning stage, two cross-entropy loss variables were defined.

$$L_{token} = \sum_{i=1}^{V} y_i \, log(\hat{y}_i)$$

Where:

- $\mathrm{y_i}$ is 1 for the actual token and 0 for all the other tokens.
- $\hat{\mathrm{y}}_{\mathrm{i}}$ is the predicted probability for the i-th token in the vocabulary taken from the predicted probability distribution vector $\hat{y}$.
- V is the vocabulary size

This loss is calculated for all tokens in the sequence and the cross-entropy loss is defined as the average across all tokens.

$$L_{CE} = \frac{1}{S}\sum_{i=1}^{S} L_{token}(i)$$

Where S is the sequence length in tokens.

The margin loss is calculated by taking a batch of pairs of protein-peptide that bind and assuming that each protein binds only to the corresponding peptide. All proteins and peptides are then converted to embedding vectors using the relevant encoders, and the Euclidean distance between each protein and all peptides in the batch is calculated.

$$D(i,j) = D_{Euclidean}(Protein_i, Peptide_j)$$

Notably, the diagonal elements D(i,i) represent the distance between protein-peptide pairs that bind (positive example distance), whereas the other elements represent distances between protein-peptide pairs that do not bind (negative example distance).

The margin function determines whether the difference between positive example distance and negative example distance exceeds minimal margin:

$$f_{margin} = max(0, D_{pos} - D_{neg} + m)$$

Where m is the minimal margin parameter (constant).

For each row in matrix D, margin function is applied, treating the diagonal elements as positives and the rest as negatives. Therefore, the first row is:

$$D_{margin}(i,j) = max(0, D(i,i) - D(i,j) + m)$$

And the margin loss is the average of $\mathrm{D_{margin}}$

$$L_{margin} = \frac{1}{n^2}\sum_{row=1}^{n}\sum_{col=1}^{n} D_{margin}(row, col)$$
